# Activation of *Bacillus thuringiensis* Cry1I to a 50 kDa stable core impairs its full toxicity to *Ostrinia nubilalis*

**DOI:** 10.1007/s00253-022-11808-2

**Published:** 2022-02-09

**Authors:** Ayda Khorramnejad, Yolanda Bel, Reza Talaei-Hassanloui, Baltasar Escriche

**Affiliations:** 1 Laboratory of Biotechnological Control of Pests, Departamento de Genética, Instituto BioTecMed, Universitat de València, Burjassot, València Spain; 2 Laboratory of Biological Control of Pest, Department of Plant Protection, College of Agriculture and Natural Resources, University of Tehran, Karaj, Iran

**Keywords:** Proteolysis, Bioassay, Binding assay, Oligomerization, Midgut juice

## Abstract

**Abstract:**

*Bacillus thuringiensis* Cry1I insecticidal proteins are structurally similar to other three-domain Cry proteins, although their size, activity spectrum, and expression at the stationary phase are unique among other members of the Cry1 family. The mode of action of Cry1 proteins is not completely understood but the existence of an activation step prior to specific binding is widely accepted. In this study, we attempted to characterize and determine the importance of the activation process in the mode of action of Cry1I, as Cry1Ia protoxin or its partially processed form showed significantly higher toxicity to *Ostrinia nubilalis* than the fully processed protein either activated with trypsin or with *O*. *nubilalis* midgut juice. Oligomerization studies showed that Cry1Ia protoxin, in solution, formed dimers spontaneously, and the incubation of Cry1Ia protoxin with *O*. *nubilalis* brush border membrane vesicles (BBMV) promoted the formation of dimers of the partially processed form. While no oligomerization of fully activated proteins after incubation with BBMV was detected. The results of the in vitro competition assays showed that both the Cry1Ia protoxin and the approx. 50 kDa activated proteins bind specifically to the *O*. *nubilalis* BBMV and compete for the same binding sites. Accordingly, the in vivo binding competition assays show a decrease in toxicity following the addition of an excess of 50 kDa activated protein. Consequently, as full activation of Cry1I protein diminishes its toxicity against lepidopterans, preventing or decelerating proteolysis might increase the efficacy of this protein in Bt-based products.

**Key points:**

• *Processing Cry1I to a 50 kDa stable core impairs its full toxicity to O. nubilalis*

• *Partially processed Cry1Ia protoxin retains the toxicity of protoxin vs O. nubilalis*

• *Protoxin and its final processed forms compete for the same functional binding sites*

**Supplementary Information:**

The online version contains supplementary material available at 10.1007/s00253-022-11808-2.

## Introduction

*Bacillus thuringiensis* (Bt) produces different proteins toxic to many insects whether at the stationary phase accumulated in the crystal inclusions (Cry and Cytolytic proteins) or secreted during the vegetative phase of bacterial growth (Adang et al. 2014). Cry1I proteins are structurally similar to the other three-domain Cry proteins sharing five conserved amino acid blocks, but they are secreted early in the stationary phase (Tailor et al. 1992; Kostichka et al. 1996; Dehury et al. 2013).

So far, many *cry1I*-type genes have been cloned and the insecticidal activity of their corresponding proteins has been demonstrated against different lepidopteran and coleopteran insect species (Tailor et al. 1992; Sekar et al. 1997; Grossi-de-Sa et al. 2007; Bergamasco et al. 2011; Zhang et al. 2013; Zhao et al. 2015). Moreover, the studied Cry1I proteins have no binding sites in common with Cry1Ab, Cry1Ac, Cry1Ah, Cry1Fa proteins in the brush border membrane vesicles of the tested insect species (Ruiz de Escudero et al. 2006; Xu et al. 2013; Zhao et al. 2015; Wang et al. 2017). Consequently, the considerable insecticidal activity of Cry1I proteins along with the lack of shared binding sites with other Cry proteins have encouraged the incorporation of the *cry1I* genes alone or pyramided with other Bt genes into the Bt transgenic crops to confer protection against lepidopteran and coleopteran pests (Lagnaoui et al. 1999; Liu et al. 2004; Selvapandiyan et al. 1998; Martins et al. 2008; de Oliveira et al. 2016; Yang et al. 2014).

Despite the importance of Bt Cry proteins as bioinsecticides, the mode of action (MOA) of Cry1A and specifically Cry1I proteins is not fully characterized, and this knowledge is crucial for designing the best functional proteins in terms of toxicity to the target pests.

In the pore formation model (the most generally accepted Cry proteins MOA), upon the proteolytic processing in the midgut of a susceptible insect, activated toxins bind to specific receptors, oligomerize, and subsequently form pores, which eventually lead to epithelial cell lysis and insect death (Adang et al. 2014).

Different biochemical and molecular analyses have provided limited information about the different steps of Cry1I proteins’ MOA. So far, the analyses have indicated that the Cry1I protoxins (with a molecular weight of about 81 kDa) are toxic for certain insect species (Tailor et al. 1992; Shin et al. 1995; Choi et al. 2000; Song et al. 2003; Ruiz de Escudero et al. 2006; Li-Ming et al. 2008; Guo et al. 2011; Zhao et al. 2015; Rodríguez-González et al. 2020). Also, C-terminal truncated Cry1I proteins were shown to have insecticidal activity (Sekar et al. 1997, Berretta et al., 2020, Guo et al. 2011). Meanwhile, the toxicity of the Cry1I trypsin-digested cores (approx. 50 kDa) has been poorly studied, and the results have shown that these activated proteins cannot retain the toxicity of their respective full-length protoxins against *O. nubilalis* (Sekar et al. 1997) or *Plutella xylostella* (Guo et al. 2009). Proteolysis is considered, in the Cry MOA, a primary step in both pore formation and signal transduction models (de Maagd et al. 2001; Pardo-López et al. 2013; Adang et al. 2014). And interruption of this activation step may lead to insect resistance (Oppert et al. 1997; Herrero et al. 2001; Talaei-Hassanloui et al. 2014; Zhang et al. 2019). Therefore, the study of the apparent peculiarity of Cry1I in terms of activation and the consequent decrease in toxicity was one of the aims of the present work.

Regarding binding, it has been described that the trypsin-activated Cry1I toxins can bind specifically to midgut receptors of susceptible hosts (Ruiz de Escudero et al. 2006; Guo et al. 2011). In general, for Cry proteins, binding to the insect receptors promotes toxin oligomerization, but in the case of Cry1Ie, it has been described that activated proteins can form oligomers spontaneously in solution (Guo et al. 2009). The oligomerization of the Cry1Ia protease-resistant core in contact with insect cells or insect BBMV has been also studied (Khorramnejad et al. 2020), whereas the ability of Cry1I protoxin to form oligomers as well as its ability to bind specifically to insect receptors has never been experimentally addressed. This information could be important since it has been shown that Cry1A proteins can have a dual mode of action in the sense that the Cry1A protoxins can also bind to insect receptors, oligomerize, and form pores in a mechanism different from that of the activated Cry1A toxins (Fabrick and Tabashnik 2007; Gómez et al. 2014; Tabashnik et al. 2015).

In this paper, we have investigated the toxicity, binding and oligomerization of the Cry1Ia protoxin, and compared them with those of the fully activated toxins obtained after treatments with trypsin or insect midgut juice, using *O*. *nubilalis* (Lep.: Crambidae) as the target pest. Also, a Cry1Ia C-terminal partially processed form has been analyzed. The results obtained will help to gain insight into the MOA of Cry1I proteins in lepidopteran insects and may help design the best strategies to combat these insect pests.

## Materials and methods

### *cry1Ia* gene isolation and cloning

The *cry1Ia38* gene (GenBank accession number MG584186) was identified and isolated from an Iranian *B*. *thuringiensis* strain (IE-1 strain) toxic for lepidopteran insect species, after a PCR-based study of the insecticidal gene content (Khorramnejad et al. 2018a). The total DNA from Bt strain IE-1 was extracted and purified following Ferrandis et al. 1999. The full-length *cry1Ia* gene comprised a 2160-bp open reading frame encoding a putative protein of 719 amino acids. The Cry1Ia sequence was submitted to the *B*. *thuringiensis* δ-Endotoxin Nomenclature Committee (https://www.bpprc.org/) and denominated Cry1Ia38. The primers used for PCR amplification of *cry1Ia38* full-length gene were Ia38-F (5′GGATCCATGAAACTAAAGAATCAAGAT3′) and Ia38-R (5′GTCGACCTACATGTTACGTTACGCTCAATC3′). The primers were designed with Primer3Plus (www.bioinformatics.nl/primer3plus) online software and synthesized by Sigma-Aldrich (Merck KGaA, Darmstadt, Germany). Amplification was performed with KAPA HiFi DNA polymerase (KAPA Biosystems, United States) in an Eppendorf Mastercycler thermal cycler (Eppendorf AG, Barkhuasenweg, Germany). PCR conditions were 3 min of initial denaturation at 95 °C followed by 30 cycles of amplification with 20 s of denaturation at 98 °C, 15 s of annealing at 63 °C, 2 min of extension at 72 °C, and an extra extension step of 10 min at 72 °C. PCR products were analyzed by 1% agarose gel electrophoresis in Tris–borate-EDTA buffer. After the amplification, the full-length PCR products were purified using NucleoSpin Gel and PCR Clean-up kit (Macherey–Nagel, Germany) and sent to be sequenced by Stab Vida (Investigação e Serviços em Ciências Biologicas, LDA, Portugal). Initially, the full-length PCR products were cloned into the cloning vector pGEM-Teasy (Promega, Madison, USA), and the pGEM-*cry1Ia38* constructs were transferred into *Escherichia coli* DHα5 competent cells. The recombinant plasmids were purified using the Nucleo-Spin Plasmid Kit (MACHEREY–NAGEL, Germany). The coding sequences were obtained after digestion with *Sal*I and *BamH*I and the sequences were confirmed by sequencing with internal primers, in both directions. The digested *cry1Ia38* gene was ligated and sub-cloned into the expression vector pET-30a( +) (Novagen, USA), and transformed into *E*. *coli* BL21 (DE3) cells for protein expression.

### Expression and purification of Cry proteins

The Cry1Ia protein expression, purification, and trypsin activation were carried out as described by Khorramnejad et al. (2018b). Protein purification was performed by Ni^2+^ affinity chromatography using a HisTrap™ FF crude column (GE Healthcare Bio-Sciences, Uppsala, Sweden) according to the manufacturer’s instructions. After purification, protein dialysis was done with SnakeSkin™ Dialysis Tubing (Thermo Scientific, Rockford, IL, USA) to exchange the elution buffer with carbonate buffer (50 mM Na_2_CO_3_, 100 mM NaCl, pH 10.5). The purity and quantity of protoxins were analyzed by 12% sodium dodecyl sulfate–polyacrylamide gel electrophoresis (SDS-PAGE) as described by Laemmli (1970) and Bradford assay (1976), respectively.

The Cry1Ab protoxin from the recombinant *E*. *coli* strain GG094-208, kindly supplied by Dr. R.A. de Maagd (Wageningen University, The Netherlands), was employed in the binding competition experiments. The Cry1Ab production was carried out as described by Sayyed et al. (2005).

### Insect rearing, midgut juice extraction, and preparation of brush border membrane vesicles

The laboratory *Ostrinia nubilalis* (Lep.: Crambidae) colony employed in this study has been maintained for at least three years at the Department of Genetics of University of Valencia (Spain). Larvae were reared on an artificial diet (Poitout and Bues 1974) and kept under controlled conditions (25 ± 1 °C, 70 ± 5% relative humidity, and with 16:8 h L:D photoperiod).

Dissected midguts from fifth instar larvae of *O*. *nubilalis* were used for midgut juice (MJ) extraction and brush border membrane vesicles (BBMV) preparation. The *O*. *nubilalis* MJ was extracted following Bel et al. (2017). BBMV were prepared based on the differential magnesium precipitation method (Wolfersberger et al. 1987; Escriche et al. 1995) from dissected midguts. The protein concentration of *O*. *nubilalis* MJ and BBMV preparations was quantified by Bradford (1976). MJ and BBMV preparations were immediately aliquoted, snap-frozen in liquid nitrogen, and kept at – 80 °C until use.

### Proteolytic activation and time course proteolysis

The Cry1Ia protoxin was activated with trypsin (trypsin from bovine pancreas, TPCK treated, Sigma-Aldrich, St. Louis, MO, USA) at a ratio of 1:10 [wt/wt] trypsin/protoxin, for 4 h at 37 °C. The proteins were quantified by densitometry with TotalLab Quant software (version 12.3, Newcastle-Upon-Tyne, UK), using bovine serum albumin (BSA) as a standard, and stored at – 80 °C until use. The TotalLab Quant software (version 12.3) was also used to assess the molecular weights of the proteins.

The Cry1Ab protoxin was activated following Sayyed et al. (2005). The activated protein was purified by anion-exchange chromatography using ÄKTA 100 explorer system (GE Healthcare, Amersham, UK) following Crava et al. 2013. Dialysis was performed to exchange the elution buffer with carbonate buffer, pH 10.5, as explained above.

Time course proteolytic processing of Cry1Ia protoxin was carried out following Bel et al. (2017). In short, Cry1Ia protoxin was digested with trypsin (trypsin from bovine pancreas, TPCK treated, Sigma-Aldrich, St. Louis, MO, USA) or *O*. *nubilalis* MJ at the ratio of 1:10 (wt trypsin/wt protoxin) at 37 °C for 2.5, 5, 15, 30, 60, 120, 180 min, and 1 day. The proteolytic processing of Cry1Ia protoxin by *O*. *nubilalis* MJ was carried out by incubation of the protein with MJ in a mass ratio of 1:10 (wt MJ/wt protoxin) at 30 °C for the same time intervals. To obtain a sample enriched in the partially processed Cry1Ia protein, protoxin was treated with trypsin at a concentration of 0.7:100 (wt trypsin/wt protoxin), for 3 h at 37 °C. For the SDS-PAGE analysis of time courses, the samples were withdrawn at different incubation times, mixed with SDS-PAGE loading buffer, immediately heated at 99 °C for 10 min, frozen in liquid nitrogen, and kept at – 20 °C until subjected to the electrophoretic analyses.

### Toxicity assays

The insecticidal activity was assessed against the first instar larvae of *O*. *nubilalis* using the surface contamination method as described elsewhere (Hernández-Martínez et al. 2008). At least seven different concentrations of the different Cry1Ia proteins were used to calculate the 50% lethal concentrations (LC_50_) values by Probit analysis (Finney 1971) using the POLO-PC program (LeOra Software, Berckely, CA, USA, 1987). The significant difference between estimated LC_50_ values was determined based on the non-overlapping of the 95% confidence intervals. At least three independent biological replicates were performed for each bioassay experiment, using 48 larvae in each tested toxin concentration. Control larvae were fed with carbonate buffer, pH 10.5. Bioassays were conducted at 25 ± 1 °C, 60 ± 5% RH, and 16:8 L/D photoperiod. The number of dead larvae was recorded after 7 days. Mortality was calculated based on the number of dead larvae.

### In vivo* binding competition*

Feeding competition experiments were performed following Jerga et al. (2019). The Cry1Ia protoxin was mixed with trypsin-activated toxin at the molar ratios 1:0, 1:1, 1:10, 1:25, and 0:25, and co-overlaid on the surface of the insect diet. The molar ratios were selected based on the LC_50_ concentration of Cry1Ia protoxin against *O*. *nubilalis* estimated in this study. In the controls, the insect food was treated with carbonate buffer, pH 10.5. For each bioassay experiment, at least three independent biological replicates were performed. Bioassays were kept at the same conditions explained above. Mortality was recorded after 7 days. The data were subjected to a one-way analysis of variance (ANOVA) and the means that were significantly different (*P* value < 0.05) were grouped by Tukey’s test.

### N-terminal and C-terminal sequences analyses

For the N-terminal amino acid sequence analyses, the protease-resistant cores of Cry1Ia protein treated with trypsin or with *O*. *nubilalis* MJ were analyzed by 12% SDS-PAGE and blotted onto a nitrocellulose membrane (Hybond™-ECL™, GE HealthCare). The proteins bound to the membrane were stained by 0.1% Coomassie blue R-250 solution in 40% methanol and 10% acetic acid for 5 min. The stained bands were excised, destained in a 50% methanol solution, and subjected to 4 to 6 cycles of N-terminal sequencing. The N-terminal amino acid residues were determined by Edman’s degradation analysis performed by Servicio de Química de Proteínas at the Centro de Investigaciones Biológicas (Madrid, Spain).

To determine the C-terminal ends of Cry1Ia fully activated proteins (activated with trypsin or *O*. *nubilalis* MJ) and Cry1Ia partially processed protein, the SDS-PAGE bands were cut and subjected to the liquid chromatography and tandem mass spectrometry (LC–MS/MS- MALDI-TOF-TOF) analysis, performed by the proteomics facility of SCSIE (Servei Central de Suport a la Investigació Experimental) at the University of Valencia, Burjassot, Spain. Based on the alignment of the detected peptide sequences, the putative cleavage sites of the activated toxins at the C-terminal end were estimated.

### In vitro* homologous and heterologous competition binding assays*

Cry1Ia protoxin and fully trypsin activated toxins were biotinylated using a protein biotinylation kit (GE HealthCare, Little Chalfont, UK) according to the manufacturer’s instructions, following Hernández-Martínez et al. (2014). The biotin-labeled protein was loaded onto a PD-10 desalting column (GE HealthCare, Little Chalfont, UK) previously equilibrated with PBS. The eluted fractions were quantified by NanoDrop 2000 spectrophotometer (ThermoFisher Scientific, Waltham, MA, USA), analyzed by 12% SDS-PAGE, and verified by Western blot as described elsewhere (Khorramnejad et al. 2020).

Homologous and heterologous binding competition experiments were performed by incubating 50 ng of the biotinylated Cry1Ia protoxin or fully trypsin activated protein, with 10 μg of *O*. *nubilalis* BBMV in the absence or presence of 200-fold of unlabeled competitors, in a final volume of 100 μl of binding buffer (PBS, 0.1% BSA). Incubations were carried out for 1 h at 25 °C. The binding reactions were stopped by centrifugation at 16,000 × *g* for 10 min at 4 °C, and the pellets were washed with 500 μl of ice-cold binding buffer. After centrifugation, the final pellets, containing BBMV and the bound biotinylated proteins, were suspended in 10 μl of the binding buffer and analyzed by 12% SDS-PAGE. The separated proteins were electrotransferred to a nitrocellulose membrane (Hybond™-ECL™, GE HealthCare). Biotinylated Cry1Ia proteins were detected by streptavidin-conjugated horseradish peroxidase (1:2000; 60 min) (GE Healthcare, Amersham, UK), and visualized by chemiluminescence using ECL™ prime western blotting detection reagent (GE Healthcare, Little Chalfont, UK) using an ImageQuant LAS400 image analyzer (GE Healthcare Bio-Sciences, Uppsala, Sweden).

### Size exclusion chromatography

Size exclusion chromatography (SEC) was carried out with an ÄKTA explorer 100 chromatography system (GE Healthcare) with a Superdex-200 10/300 GL column (GE Healthcare Life sciences, Uppsala, Sweden). Prior to use, the column was calibrated with the following mix of protein standards: 44 kDa ovalbumin (4 mg/ml), 75 kDa conalbumin (3 mg/ml), 158 kDa aldolase (4 mg/ml), 440 kDa ferritin (0.3 mg/ml), and 669 kDa thyroglobulin (5 mg/ml). The column was equilibrated with carbonate buffer, pH 10.5. The status of Cry1Ia protoxin and proteolytically processed toxins in terms of monomeric versus oligomeric forms was determined based on the molecular weight of the chromatographic eluted peaks.

The presence of the oligomeric forms of the protein was checked using semi-native PAGE conditions, which consisted of treating the samples with a loading buffer without SDS and β-mercaptoethanol. Additionally, the samples were heated at 50 °C (instead of 99 °C) before loading them into the gel.

### BBMV oligomer promotion assay

The formation of Cry1Ia oligomeric structures promoted by *O*. *nubilalis* BBMV was studied following Khorramnejad et al. (2020) with slight modifications. Briefly, 5 µg of Cry1Ia protoxin or fully trypsin activated toxin were incubated with 2 µg of *O*. *nubilalis* BBMV in a final volume of 50 µl of carbonate buffer (pH 10.5), for 1 h at 37 °C. For controls, Cry1Ia protoxin or trypsin activated toxin was incubated in the same conditions as a treatment but without BBMV. The reaction was stopped by adding 1 mM PMSF and incubating on ice for 2–3 min. The BBMV with bound proteins were separated by centrifugation at 21,100 × *g* for 30 min at 4 °C. The supernatant containing the unbound protein was also analyzed. The pellet was washed with 100 µl of ice-cold carbonate buffer (pH 10.5) and centrifuged at 21,100 × *g* for 15 min at 4 °C. The final pellet was resuspended in 10 µl of the same buffer, mixed with sample loading buffer, and incubated at 50 °C for 3 min. Controls of only BBMV (2 µg) or only Cry1Ia incubated without BBMV were also loaded into the SDS-PAGE gel. The proteins were separated by 10% SDS-PAGE, electrotransferred onto PVDF Western blot membrane, and detected with polyclonal anti-Cry1Ia (1/10,000; 60 min) followed by anti-rabbit IgG-conjugated horseradish peroxidase (1/20,000; 60 min). The sensitivity of the polyclonal anti-Cry1Ia antibody had been previously tested by dot blot, showing that the antibody could detect precisely both the protoxin and the fully activated toxin at a concentration as low as 0.5 ng/µl, 1000 times less than the concentration of protein used in the oligomerization assays. The membrane was visualized by chemiluminescence using ECL™ prime western blotting detection reagent (GE Healthcare, Little Chalfont, UK) using an ImageQuant LAS400 image analyzer (GE Healthcare Bio-Sciences, Uppsala, Sweden). The oligomerization assays for each Cry1Ia protein were repeated at least three times.

## Results

### Processing of the Cry1Ia protoxin with trypsin or O. nubilalis MJ

**Fig. 1:**
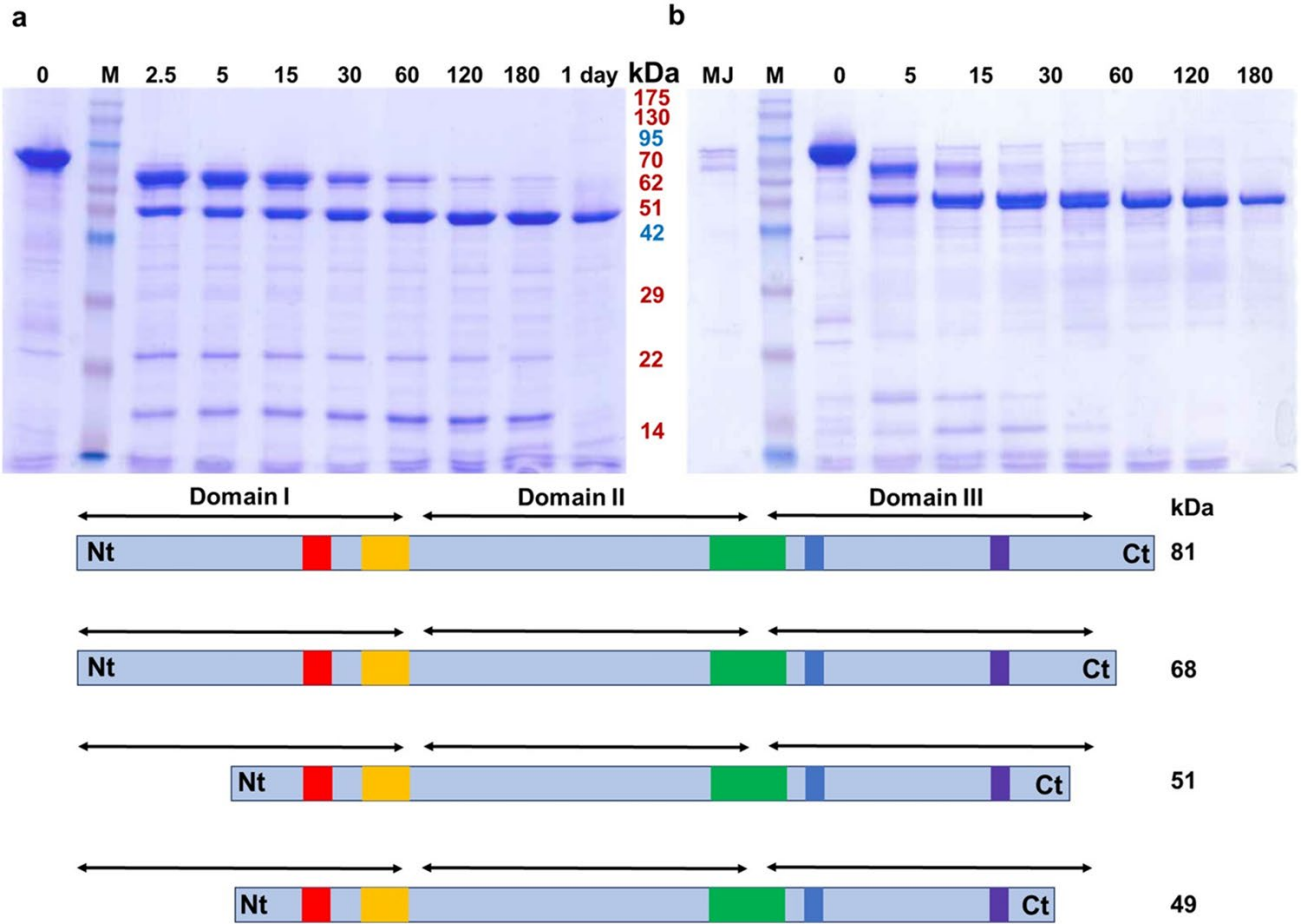
Proteolytic processing of Cry1Ia. **a** Time course activation with trypsin. **b** Time course activation with *O*. *nubilalis* MJ. The times (minutes) at which the processing status was analyzed are indicated at the top of each lane. Lanes 0: Cry1Ia protoxin. Lane MJ: *O*. *nubilalis* MJ at the same concentration used in the assay. Lanes M: molecular mass marker (Pink pre-stained protein ladder, Nippon Genetics, Düren, Germany). **c** Schematic diagram that shows the Cry1Ia protoxin and its proteolytic activation products

The time course incubation of the purified Cry1Ia protoxin with trypsin or with *O*. *nubilalis* MJ is presented in Fig. 1. Although the similar band pattern, slight differences in proteolytic processing were observed between both treatments.

In vitro activation of Cry1Ia protoxin with trypsin rendered a partially processed Cry1Ia product of about 67 kDa, and another band of about 51 kDa, corresponding to the fully activated protein (Fig. 1a). Both bands were observed almost immediately (2.5 min) after starting the treatment. After 60–120 min of treatment, the 67 kDa band disappeared, whereas the final 51 kDa stable core accumulated over time. In vitro activation of Cry1Ia protein with *O. nubilalis* MJ resulted in a very similar pattern (Fig. 1b). But differently to what occurred after trypsin treatment, the concentration of the approx. 51 kDa band decreased over time and a band of a slightly lower molecular weight appeared and accumulated, resulting in a stable core of about 49 kDa (Fig. 1b). In the assay conditions used, the ~ 67 kDa intermediate form was found to be less persistent after MJ processing.

To determine whether the trypsinized Cry1Ia toxin could be further processed to the stable core obtained after protease activation with *O*. *nubilalis* MJ, the Cry1Ia protoxin was incubated first with trypsin for 2 h and then with *O*. *nubilalis* MJ (Supplementary Fig. 1). The results showed that after 15 min, the approx. 51 kDa band obtained after trypsin treatment was processed to the smaller stable band of about 49 kDa indicating further processing by other midgut enzymes.

Based on the Edman’s degradation results, the N-terminal sequences of trypsinized and MJ activated Cry1Ia toxins are identical, starting after Arg^155^ residue. The sequence of Ser^1^-Val^2^-Val^3^-Lys^4^-Ser^5^-Gln^6^ are the first six N-terminal predicted amino acids for both of them. Therefore, the results indicated that the differences in Cry1Ia protein processing by trypsin and MJ proteases were due to the ability of *O*. *nubilalis* gut proteases to further process the protein at the C-terminal end.

The fully trypsinized and MJ activated Cry1Ia toxins as well as the ~ 67 kDa partially processed form were subjected to LC–MS/MS analysis. The alignment of the detected peptide sequences helped us to predict the putative cleavage sites of the processed toxins at the C-terminal end. Based on the obtained results, the trypsin- and MJ-fully activated toxins were most likely cleaved C-terminally at Arg^670^ and Lys^659^ amino acids, respectively, which indicated that these two processed fragments differ in 11 amino acids at their C-terminal ends. The LC–MS/MS analysis of the ~ 67 kDa partially processed protein highlighted that this intermediate form conserved the N-terminal end of the protoxin but it had been processed in the C-terminal end as the fully processed partners (Fig. 1c).

### Insect toxicity

Bioassay experiments with Cry1Ia protoxin and proteolytically activated toxins obtained after treatment with trypsin or *O*. *nubilalis* MJ were performed, using *O*. *nubilalis* first instar larvae. The results are shown in Table 1 and indicate that *O*. *nubilalis* first instar larvae were susceptible to the Cry1Ia protoxin, with an LC_50_ value of 56 ng/cm^2^. While, at the LC_50_ level, toxicity was still high when the larvae were intoxicated with the partially processed protein but it decreased significantly (about 40- and 16-fold) when the larvae were treated with fully trypsinized (LC_50_ = 2197 ng/cm^2^) or MJ activated (LC_50_ = 880 ng/cm^2^) toxins, respectively. Based on our findings, fully proteolytic processing of Cry1Ia protoxin to a stable core, whether by trypsin or midgut proteases treatment, resulted in a significant loss of toxicity against *O*. *nubilalis* larvae. The lower activity of the partially processed protein compared to the one of protoxin can be explained by the presence of some fully activated protein in the partially processed protein preparations.

**Table 1:** LC_50_ values of protoxin, partially trypsinized, fully trypsinized and fully MJ activated forms of Cry1Ia protein against *O. nubilalis* first instar larvae

Protein	LC_50_ (ng/cm^2^)	Confidence Intervals (95%)		Slope ± SE
		Lower	Upper	
Protoxin	56	40	76	3 ± 0.6
Partially trypsinized	140	99	196	2.1 ± 0.2
Fully trypsinized	2197	1508	4544	2 ± 0.4
Fully MJ activated	880	665	1251	1.6 ± 0.2

Regarding fully processed Cry1Ia proteins, MJ activated Cry1Ia was about 2.5 times more toxic (at the LC_50_ level) than the trypsinized toxin. This was an unexpected result since the activation experiments showed that fully trypsin activated form was further processed by MJ enzymes to the same final product (Supplementary Fig. S1) and therefore the same toxicity was expected.

### Study of the oligomeric state of Cry1Ia proteins in solution and toxicity tests

**Fig. 2:**
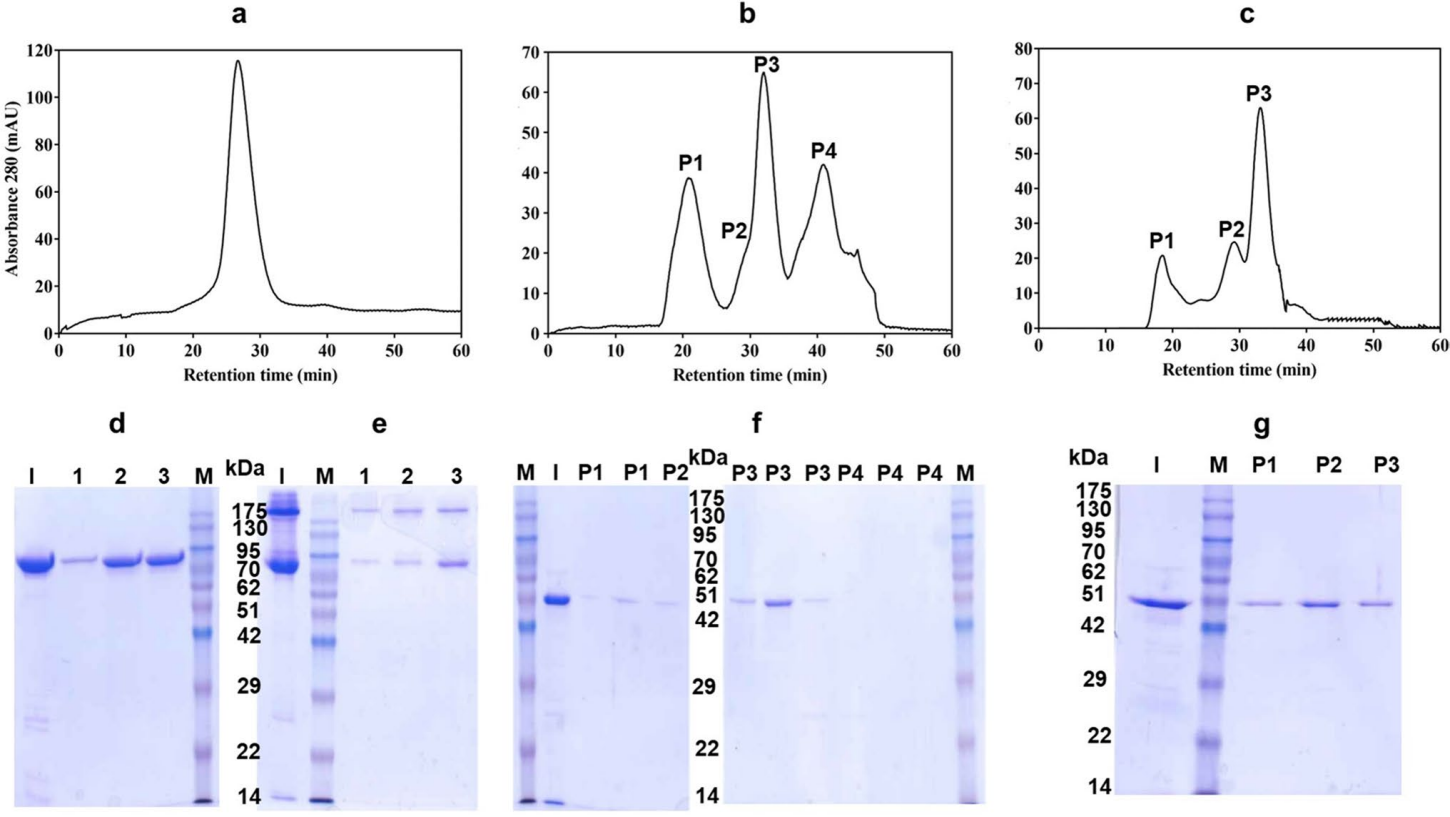
Size exclusion chromatography analysis of Cry1Ia protoxin (**a**), fully trypsin activated (**b**), and fully MJ activated protein (**c**). The Cry1Ia protoxin and the fully digested proteins were loaded into the Superdex-200 10/300 GL column. Following SEC analysis, the elution fractions corresponding to the protoxin peak were analyzed by SDS-PAGE (**d**) and semi-native-PAGE (**e**). The eluted fractions corresponding to the peaks observed after SEC of the trypsin activated and MJ activated proteins were also analyzed by SDS-PAGE (panels **f** and **g**, respectively). M, Molecular weight markers in kDa, I, sample loaded into the column

To determine the status of Cry1Ia protoxin in solution, the purified and solubilized protoxin was subjected to size exclusion chromatography (SEC). The protoxin eluted as a single peak at 26.8 min, which corresponded to a molecular weight of ~ 177 kDa (Fig. 2a), that fitted with a dimeric form. The SDS-PAGE analysis of the protein in this peak showed a single band with a molecular weight of about 80 kDa that corresponded to Cry1Ia protoxin monomer (Fig. 2d). While, when the elution peak was subjected to semi-native electrophoresis conditions, the results clearly showed the presence of two bands, one corresponding to the MW of the monomer and the other of molecular weight higher than 175, showing the existence of the oligomeric forms (Fig. 2e).

The trypsin fully processed Cry1Ia subjected to SEC eluted in three different peaks at 20.92, 32.02, and 40.90 min (Fig. 2b) which corresponded to protein aggregates (P1 in Fig. 2b) with an approximate molecular weight of ~ 630 kDa, monomers (P3 in Fig. 2b) with a molecular weight of ~ 51 kDa and dimers (shoulder labeled as P2 in Fig. 2b) with a molecular weight of ~ 107 kDa. Ultimately, the last peak (P4 in Fig. 2b) corresponded to the elution of very small peptides of less than 5 kDa. The fractions from the P1, P2, and P3 peaks were analyzed by SDS-PAGE, and a single band of ~ 51 kDa was observed in all samples (Fig. 2f).

The SEC analyses of the MJ fully processed Cry1Ia resulted in the appearance of three different peaks (Fig. 2c). The first peak (P1), with a molecular weight higher than 630 kDa, corresponded to toxin aggregates. The second peak (P2) appeared at 29.2 min and corresponded to a molecular weight of a toxin dimer (~ 100 kDa). The following peak (P3) corresponding to the MW of the monomer, eluted at 33 min. A sample of each one of the eluted peaks was analyzed by SDS-PAGE, showing a single band corresponding to the monomer in all cases (Fig. 2g).

Summarizing, the results of the SEC indicated that Cry1Ia protoxin remains in the form of a dimer in the solution. While, after full protein activation, Cry1Ia toxins were found as aggregated, dimeric, and monomeric forms being monomers the predominant forms.

**Fig. 3:**
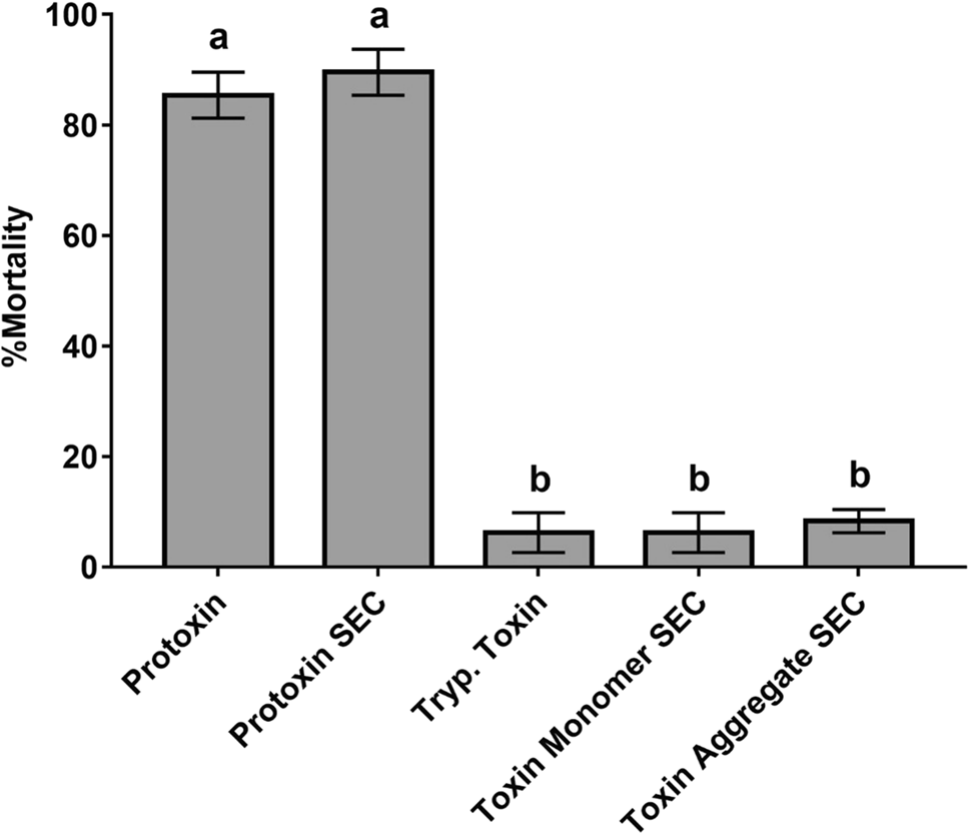
Insecticidal activity of Cry1Ia protoxin and fully trypsin activated protein before and after Size Exclusion Chromatography (SEC). The toxicities were assessed using a single concentration of 100 ng/cm^2^ against *O*. *nubilalis* first instar larvae. The columns represent the averages of the percentages of mortality (± standard error). The lowercase letters above the bars show significant differences between treatments

The insecticidal activity of the Cry1Ia proteins eluted after SEC (protoxin dimers, as well as aggregates and monomers of the fully trypsinized protein) was assessed against *O. nubilalis* first instar larvae. The bioassays were performed at a single concentration of 100 ng/cm^2^, and the results (Fig. 3) showed that protoxin dimers retain the same activity as protoxin before SEC. The highest toxicities were observed in the protoxin treatment and, in agreement with the results shown in Table 1, the insecticidal activities of the trypsin fully processed protein forms obtained after SEC separation were significantly lower. No statistically significant differences were observed between the toxicity of the fully activated protein before SEC, and the SEC monomers or aggregates.

### Oligomerization of Cry1I proteins promoted by O. nubilalis BBMV

**Fig. 4:**
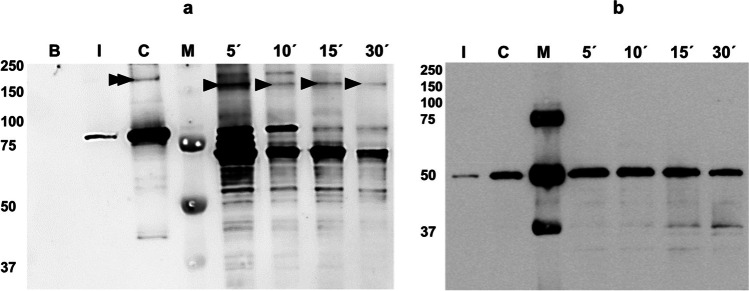
Promotion of Cry1Ia oligomers after incubation of Cry1Ia protoxin (**a**) and fully trypsinized protein (**b**) with *O*. *nubilalis* BBMV. Lanes M: Precision Plus Protein™ Dual Color molecular mass marker (Bio-Rad), molecular masses are indicated in kDa. Lane B: 2 µg of *O*. *nubilalis* BBMV. Lanes I: Cry1Ia protoxin or Cry1Ia fully trypsinized protein (10 ng) before starting incubations. Lanes C: Control of Cry1Ia proteins incubated in absence of BBMV. Lanes 5’, 10’, 15’and 30’: Cry1Ia proteins incubated with *O*. *nubilalis* BBMV at different times (5, 10, 15, and 30 min, respectively) after starting the incubation. The arrowheads point to the protoxin intermediate fragment dimer and the double arrowhead points to the dimer of the protoxin

Western blot analyses revealed that *O*. *nubilalis* BBMV promoted tetrameric oligomers neither in Cry1Ia protoxin nor in trypsin activated toxin (Fig. 4 a and b). In agreement with SEC results, in the protoxin control (in which the protein had been incubated without the presence of BBMV), besides the 80 kDa full-length protoxin, a signal with a molecular weight of about 200 kDa corresponding to a putative protoxin dimer can be also observed (Fig. 4a, lane C). Five minutes after starting the incubation of Cry1Ia protoxin with *O*. *nubilalis* BBMV, highlighting the high processing activity of BBMV, different bands were observed in the pelleted fraction and therefore, associated with the BBMV: apart from the band of about 80 kDa corresponding to the full-length protoxin, a band of ~ 67 kDa corresponding to the partially processed protoxin appeared as well a band of about 50 kDa corresponding to the fully processed protein. It is noteworthy to mention that a band with a molecular weight of about 150 kDa (lower than the protoxin dimer) most probably corresponding to dimers of the partially processed form, appeared at the same time as the partially processed fragment, indicating that the formation of a dimer of this intermediate fragment was promoted by the *O*. *nubilalis* BBMV. The presence of the intermediate fragment dimer was evident after 5 min of incubation, but its abundance, as well as the amount of full-length protoxin, the partially processed fragment, and the protease-resistant core, all decreased as the incubation progressed, most probably due to the BBMV concentration used. Moreover, the results show that the full-length Cry1Ia protoxin was bound to *O*. *nubilalis* BBMV in both monomeric and dimeric forms (Fig. 4a, lanes 5’ and 10’).

Following incubation of the trypsin fully activated protein with *O*. *nubilalis* BBMV, no oligomeric structure (dimer or tetramer) could be observed (Fig. 4b). The ~ 51 kDa band observed in the control lane corresponds to the monomer of the trypsin-resistant core (Fig. 4b, lane C). After incubation with BBMV, this monomeric form remains bound to BBMV with time.

### Competition assays

**Fig. 5:**
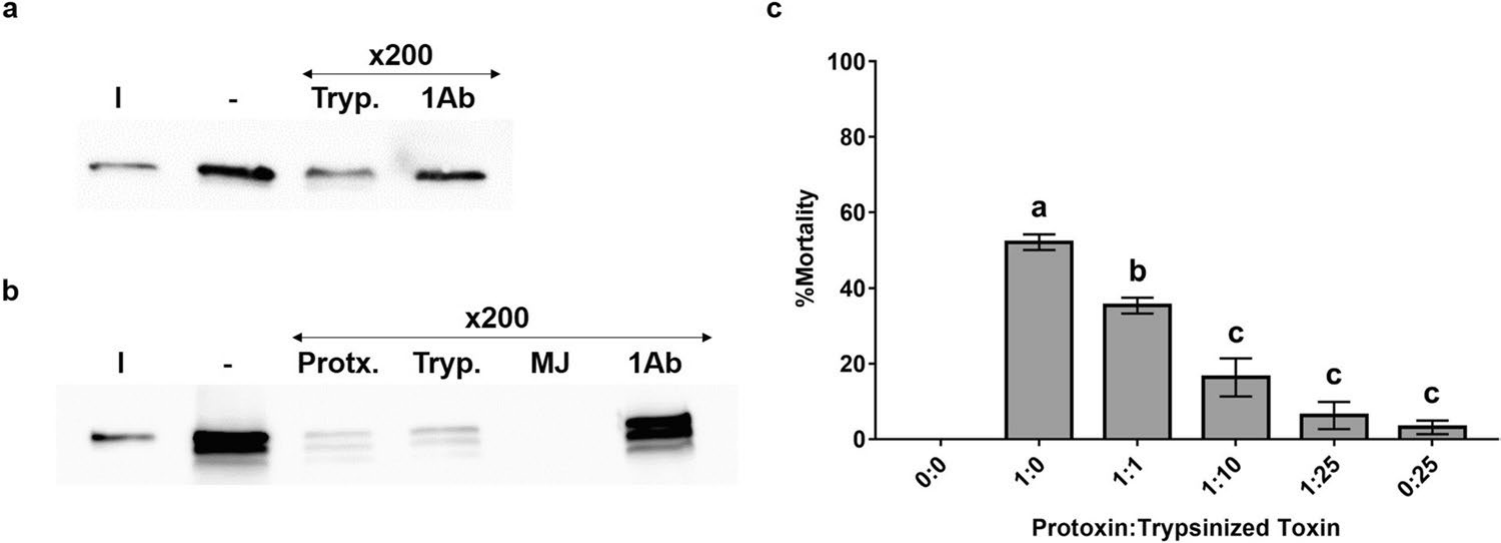
In vitro and in vivo competition experiments of Cry1Ia proteins. Biotin-labeled Cry1Ia trypsin activated protein (**a**) or protoxin (**b**) were incubated with *O*. *nubilalis* BBMV in the absence ( −) or the presence of 200-fold excess of the unlabelled competitors indicated on top of each lane. I: labeled proteins not submitted to competition experiments. Protx: Cry1Ia protoxin; Tryp: fully trypsin activated Cry1Ia; MJ: fully MJ activated Cry1Ia; 1Ab: trypsin activated Cry1Ab. Panel c: Insecticidal activity of Cry1Ia protoxin and toxin co-supplied at different molar ratios, against *O*. *nubilalis* first instar larvae. The Cry1Ia protoxin was mixed with trypsinized toxin at the molar ratios of protoxin:toxin indicated at the bottom of each column. In the control (0:0), larvae were treated with carbonate buffer, pH 10.5. Values represent the average of mortality percentages (± standard error). The lowercase letters above the bars show significant differences between treatments

In vitro, binding assays showed that Cry1Ia proteins (whether as fully trypsinized protein or protoxin) bind specifically to *O*. *nubilalis* BBMV as the binding of biotinylated Cry1Ia was displaced by the addition of 200-fold excess of the homologous unlabeled forms (Fig. 5a and b).

The results of the heterologous competition assay showed that trypsin or MJ fully activated Cry1Ia proteins competed for the Cry1Ia protoxin binding sites on *O*. *nubilalis* BBMV (Fig. 5b). Also, as a control, heterologous competition assays were performed with activated Cry1Ab (Fig. 5a and b). As expected, the results showed that 200-fold excess of Cry1Ab did not displace Cry1Ia from its binding sites in the *O*. *nubilalis* BBMV confirming that these proteins do not share binding sites.

The in vivo competition experiments were performed to investigate if the trypsin fully activated Cry1Ia could decrease the toxicity of Cry1Ia protoxin against *O*. *nubilalis* as a consequence of sharing binding receptors. The results obtained are summarized in Fig. 5c. A dose of 56 ng/cm^2^ of Cry1Ia protoxin (LC_50_ value) caused an average of 52 ± 2% mortality of *O*. *nubilalis* first instar larvae. At the 1:1 equivalent molar ratio of protoxin:toxin, mortality decreased significantly to about half of the previous value. Consistently, a decrease of Cry1Ia protoxin insecticidal activity was observed in the ratios of 1:10 and 1:25. While 25-fold molar excess (847 ng/cm^2^) of trypsinized Cry1Ia toxin had no significant effect on *O. nubilalis* larvae (3 ± 2% mortality). In conclusion, the result of the in vivo competition assay showed that the toxicity of Cry1Ia protoxin decreased following the addition of an excess of fully trypsinized Cry1Ia toxin, which was in agreement with the results obtained from the in vitro competition experiments described above and confirmed that they share the same functional binding sites in the insect midgut*.*

## Discussion

Cry1I proteins in their protoxin form have a three-domain structure and contain five conserved blocks similar to the typical Bt Cry1 proteins (Tailor et al. 1992; Sekar et al. 1997). But the Cry1I protoxins have a smaller size compared to the rest of Cry1 proteins. This is because they have a shorter C-terminal end since Cry1I proteins are truncated, and after the fifth amino acid block they broaden only 76 amino acids (Sekar et al. 1997). Other special features of Cry1I proteins are that they are secreted at the stationary phase, their genes are usually silent in Bt strains, and they have dual activity against lepidopteran and coleopteran pests (Tailor et al. 1992; Choi et al., 2000). Despite the potentials of Cry1I proteins in the effective control of insect pests, their mode of action is hardly known. Therefore, to provide more insight into the Cry1I protein’s mechanism of action, in the present study, we have attempted to compile and expand the current knowledge about Cry1I proteins by investigating the toxicity, oligomerization, and binding of Cry1Ia protoxin compared to those of the Cry1Ia partially activated and fully activated forms, using the lepidopteran *O*. *nubilalis* as target pest.

The proteolytic activation of Cry1Ia protein after exposure to trypsin or *O*. *nubilalis* MJ proteases yielded two major fragments of approximately 67 and 50 kDa at short times that ultimately resulted in a single final stable toxic core of about 50 kDa. The final processed core resulting from trypsin treatment was slightly bigger than that of MJ treated toxin. Data obtained by N-terminal sequencing indicated that Cry1Ia protein is processed at serine residue 156 at the N-terminal end coinciding with what was shown earlier (Seko et al. 1997). The first six N-terminal amino acids after Ser^156^ are conserved among the Cry1I members as found out after alignment performed in this study (data not shown) and by Ruiz de Escudero et al. (2006). Therefore, the difference found between the molecular weight of trypsinized Cry1Ia and MJ treated toxin was because gut proteases processed the C-terminal end of Cry1Ia protein to a different extent. Then, the in vitro long-term complete activation of Cry1Ia protoxin with trypsin did not mimic the activation process that happened by the mixture of proteases in *O*. *nubilalis* midgut. On the other hand, the LC–MS/MS-MALDI-TOF-TOF analysis of the 67 kDa partially processed protein showed that this intermediate form is protoxin only cleaved by trypsin at its C-terminal end as the fully processed partners.

The insecticidal activity of some Cry1I protoxins had been previously investigated against the lepidopteran *O*. *nubilalis* (Sekar et al. 1997; Zhao et al. 2015; Khorramnejad et al. 2020). In the present study, the toxicity of the newly described Cry1Ia38 protein was assessed against *O*. *nubilalis,* and based on our results the Cry1Ia38 protoxin showed about 5 times higher activity than Cry1Ia7 (Khorramnejad et al. 2020) with an LC_50_ of 56 ng/cm^2^. The toxicity of Cry1Ia protoxin was much higher than that obtained using trypsin or MJ fully activated proteins (LC_50_ values of 2197 and 880 ng/cm^2^, respectively). The proteolysis processing significantly decreased the insecticidal activity of Cry1Ia protein, indicating that the full activation of Cry1I proteins could be assumed as a degradation process from the lepidopteran toxicity point of view. Indeed, so far, the entomocidal potency of Cry1I proteins has been assessed mostly using purified protoxins (Shin et al. 1995; Choi et al. 2000; Boncheva et al. 2006; Ruiz de Escudero et al. 2006; Li-Ming et al. 2008; Guo et al. 2009; Zhao et al. 2015; Rodríguez-González et al. 2020; Berretta et al. 2020), *E*. *coli* cell lysates (Bergamasco et al. 2013), or inclusion bodies (Dammak et al. 2010). Although it has been assumed that Cry1I mode of action included an activation step to a stable active core similar to the other Cry proteins, few studies have investigated the insecticidal activity of the activated Cry1I as a stable ~ 55–50 kDa core. Previous studies based on the non-overlapping 95% confidence intervals (Sekar et al. 1997) or comparing the expected toxicity based on the molar ratios of protoxin to the toxin (Guo et al. 2009) had shown that in lepidopteran, the toxicity of trypsin-activated toxin was significantly lower than the one of the protoxin. On the other hand, bioassays performed with the partially activated, unstable ~ 67 kDa intermediate product obtained after partial trypsin activation, or studies done with C-terminal truncated Cry1I proteins resulted in toxicities similar to the ones obtained with the full-length protoxins or improved the toxicity to the tested insect species (Craveiro et al. 2010; Guo et al. 2011; Feng et al. 2015; Berretta et al. 2020). Only the study of Boncheva et al. 2006 showed improved toxicity of activated Cry1Ia compared with protoxin for *Cydia pomonella*, but the activated form had a molecular weight similar to the usual molecular weight of other Cry1 trypsin-activated proteins (about 65–70 kDa) which, for Cry1I, corresponds with the partially processed form. Our findings confirm that the ~ 50 kDa stable core obtained upon incubation with trypsin or MJ did not retain the insecticidal activity of Cry1Ia protoxin against *O*. *nubilalis* larvae. Therefore, Cry1I protoxins or partially processed protoxins are the ones that provoke the highest insecticidal activity in lepidopteran pests.

In the pore formation model (the most widely accepted model in Cry MOA), after activation, Cry proteins bind to receptors in the brush border membrane of midguts which leads to a further cleavage of the helix α1 in Domain I that promotes protein oligomerization (most probably in the form of tetramers). This has been described as a crucial step in toxicity, necessary for insertion into cell membrane to exert their toxicity (Soberon et al. 2007; Pardo-López et al. 2013). Regarding Cry1I proteins, it had been described that after full activation of Cry1Ie to the 50 kDa form, about 40% of the protein spontaneously forms aggregates as well as a little amount of dimers, being the aggregates the more stable (and by far less toxic) form (Guo et al. 2009). In the present study, we have studied the oligomeric structures of Cry1Ia protoxin, trypsinized and MJ-activated proteins, in solution and also after incubation with *O*. *nubilalis* BBMV. As a result, the Cry1Ia protoxin remained as a dimer in the solution (carbonate buffer, pH 10.5). While, in agreement with Guo et al. (2009), the protease-resistant cores have a tendency to remain as monomeric forms but also form dimeric structures and to aggregate. Moreover, according to what was described by these authors, in our study, the toxicity of the aggregates and monomers of the trypsinized toxin were significantly lower than the protoxin.

In this study, the formation of the oligomeric structures following incubation of Cry1Ia protoxin and toxin with *O*. *nubilalis* BBMV was also assessed. As a result, *O*. *nubilalis* BBMV promote the formation of tetrameric structures (as the most known and conventional Cry protein oligomers), neither in protoxin nor in trypsin activated toxin. In agreement with SEC results, protoxin dimers were observed in controls of Cry1Ia protein in solution, not incubated with BBMV. But it is noteworthy to mention that we have found that *O*. *nubilalis* BBMV promoted the formation of dimeric structures of the ~ 67 kDa unstable protoxin intermediate. These dimers were found associated with the BBMV. Regarding the fully activated Cry1Ia proteins, the results obtained are in agreement with previous studies, which showed that biotin-labeled and trypsinized Cry1Ia toxin did not oligomerize following incubation with susceptible lepidopteran BBMV or non-susceptible insect cell line (Khorramnejad et al. 2020).

The determination of the three domains of Cry1I proteins based on structural analyses (Grossi-de-Sa et al. 2007; Dehury et al. 2013; Feng et al. 2015) enables us to explain the lack of the typical Cry1 oligomeric structures. According to these structural analyses, Cry1I protein domain I include amino acids Met^1^ to Thr^280^ (Grossi-de-Sa et al. 2007; Dehury et al. 2013; Feng et al. 2015). Based on our N-terminal sequencing results and Sekar et al (1997), the ~ 50 kDa Cry1Ia lacks 155 amino acids of the N-terminal end in domain I, which includes the helices α1-α4 and part of helix α-5 in domain I. Structure–function studies have shown that helices α1-α5 play a crucial role in membrane insertion, oligomerization, and pore formation of Cry proteins (Uawithya et al. 1998; Aronson and Shai, 2001; Jiménez-Juárez et al. 2007; Girard et al. 2009). Therefore, the absence of these structures could explain the absence of Cry1Ia tetrameric structures promoted by *O*. *nubilalis* BBMV.

Homologous competition binding assays showed that the binding of both protoxin and fully trypsinized Cry1Ia to *O*. *nubilalis* BBMV was specific. The results of heterologous binding assays showed that Cry1Ia fully activated proteins compete with Cry1Ia protoxin for the same binding sites on *O. nubilalis* BBMV. This is consistent with the results of the in vivo binding competition assays that showed the toxicity of Cry1Ia protoxin was impaired following the addition of the 50 kDa trypsinized Cry1Ia. The implication of this finding is crucial since the processing of the protein inside the insect midguts is a dynamic process and therefore the toxicity of Cry1Ia in lepidopterans can be highly compromised as the 50 kDa stable core concentration increases in the midgut environment, decreasing its effectivity. Apparently, designing a Cry1I protein that could avoid N-terminal activation could improve the toxicity against lepidopterans. This was attempted in this study by designing two mutants with altered N-terminal trypsin cleavage sites. The cleavage sites were predicted by both the ExPASy Peptide Cutter tool (Gasteiger et al. 2005), in silico analysis of the most exposed trypsin cleavage sites in the Cry1Ia structure, and based on N-terminal sequencing results. The mutants were successfully generated and expressed, but unfortunately, they could not achieve the goal of circumventing processing (Supplementary Fig. S2), probably due to alternative processing sites.

Additionally, the results of our work confirmed that neither Cry1Ia protoxin nor the ~ 50 kDa trypsinized toxin shared binding sites with Cry1Ab in *O*. *nubilalis* BBMV. The lack of shared binding sites and the high levels of toxicity of Cry1Ia and Cry1Ab proteins to *O*. *nubilalis* (Khorramnejad et al. 2020) indicates that these proteins could be good candidates to be pyramided in the Bt transgenic maize. Indeed, other *cry1I* genes have been proposed to be pyramided with other *cry1* genes in the transgenic crops since they do not share common binding sites in the tested insect species (Zhang et al. 2013; Zhao et al. 2015).

The structural analyses indicate that Cry1Ia protoxin shares domains II and III with fully activated toxins. Cry1Ia domain II has been introduced as a crucial determinant for toxicity of Cry1Ba/Cry1Ia hybrid toxins against the Colorado potato beetles and domains II and III might be considered important in receptor binding (Naimov et al. 2001). Accordingly, our binding experiments showed the specific binding of the fully processed Cry1Ia to *O*. *nubilalis* BBMV (Fig. 5), while this binding did not lead to full toxicity. As well, the possible role of Cry1Ia12 domain I in the toxin insertion has been suggested based on the molecular modeling analyses alongside the toxicity of the Cry1Ia variants against *Telchin licus licus* (Craveiro et al. 2010). Moreover, the sequence and structural alignments of Cry1Id with its closest Cry proteins’ homologues suggested that Cry1Id domain I can be involved in membrane penetration and pore formation (Dehury et al. 2013). Similarly, domain I of Cry1Ie protein can insert into lipid monolayers, whereas maintaining this penetration needed all three domains (Guo et al. 2014). These results besides our findings point to the importance of Cry1Ia domain I in toxicity to lepidopterans. Indeed, domain I is present in the protoxin and the partially processed ~ 67 kDa form which both shows high toxicity to *O*. *nubilalis*, but is mostly lost during the proteolytic processing that leads to the less toxic 49–51 kDa Cry1Ia stable cores.

Based on our results, Cry1I protoxin can bind to *O*. *nubilalis* BBMV specifically, and this binding leads to the formation of a dimeric structure of a partially processed protoxin fragment of ~ 67 kDa. Whereas, fully processed Cry1Ia could bind to the same *O*. *nubilalis* BBMV functional receptors, but this binding leads to neither oligomerization nor full toxicity. Therefore, our results suggest that the full activation process might not be an essential step in the mode of action of Cry1I proteins against lepidopteran pests, and attempts to maintain or increase the half-life of the partially processed protein could lead to higher levels of toxicity against these insects. Whether this could affect or not the Cry1Ia coleopteran dual activity should be further investigated. This work might help to improve pest management strategies, modifying *cry1I* genes in Bt crops in order to improve the functional characteristics of the toxin related to stability, binding ability, and insecticidal activity.

## Supplementary Information

Below is the link to the electronic supplementary material.

Supplementary file1 (PDF 275 KB)

## Data Availability

Available on reasonable request.
