# Supplementary material for: Activation of Bacillus thuringiensis Cry1I to a 50 kDa stable core impairs its full toxicity to Ostrinia nubilalis
Source: Appl Microbiol Biotechnol. 2022 Feb 9;106(4):1745–58. doi: 10.1007/s00253-022-11808-2 (PMC8882101; doi:10.1007/s00253-022-11808-2)
Supplement: Supplementary file 1 — Supplementary file1 (PDF 275 KB) [file 253_2022_11808_MOESM1_ESM.pdf]

Applied Microbiology and Biotechnology

Supplementary Information

Activation of *Bacillus thuringiensis* Cry1I to a 50-kDa stable core impairs its full toxicity to *Ostrinia nubilalis*

Ayda Khorramnejad[1,2], Yolanda Bel[1*], Reza Talaei-Hassanloui[2], Baltasar Escriche[1*]

[1]Laboratory of Biotechnological Control of Pests, Instituto BioTecMed, Departamento de Genética, Universitat de València, Burjassot, València, Spain

[2]Laboratory of Biological Control of Pest, Department of Plant Protection, College of Agriculture and Natural Resources, University of Tehran, Karaj, Iran

*** Corresponding authors:**
Baltasar Escriche: baltasar.escriche@uv.es
Yolanda Bel: yolanda.bel@uv.es

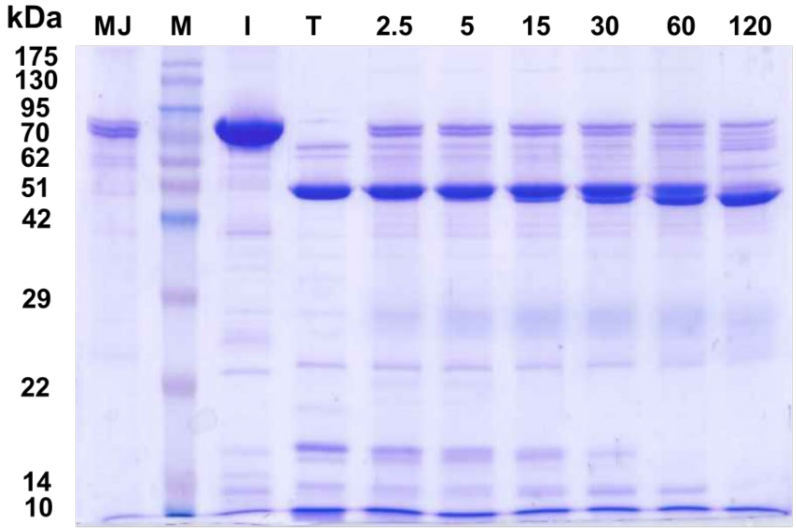

Supplementary Fig. S1. Time course of proteolytic processing of trypsinized Cry1Ia with *O. nubilalis* MJ. The trypsinized Cry1Ia toxin was incubated with *O. nubilalis* MJ (ratio of 1:5 [wt MJ protease/ wt protein]). Aliquots were taken at the times (minutes) indicated on top of each lane. Lane MJ: *O. nubilalis* MJ at the concentration used in the assay. Lane I: Cry1Ia protoxin. Lane T: Trypsin treated Cry1Ia at the ratio of 1:10 (wt trypsin/wt protoxin) for two hours. Lane M: molecular mass marker (Pink pre-stained protein ladder, Nippon genetics).

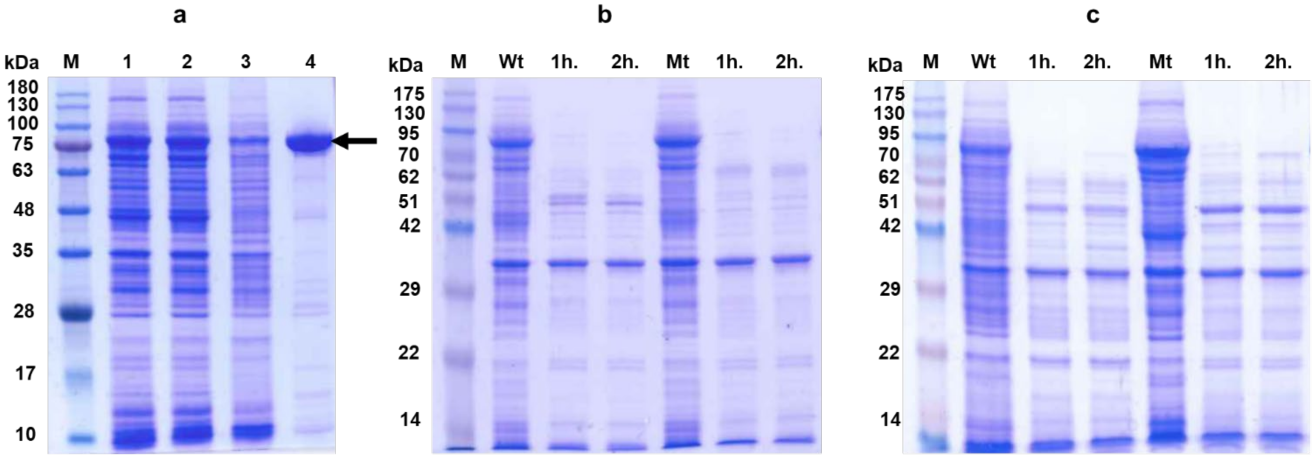

Supplementary Fig. S2. Expression (a) and trypsin treatment (b and c) of Cry1Ia wild-type and mutant proteins. Cry1Ia mutants with a double mutation of R155A+K159A and a triple mutation of R121A+K123A+K129A were constructed by site-directed mutagenesis based on the in silico prediction of trypsin cleavage sites by ExPASy Peptide Cutter tool (Gasteiger et al. 2005), analyzing the exposed trypsin cleavage site, and N-terminal sequencing results. Panel a; Lane 1: Mutagenic Cry1Ia with a double mutation of R155A+K159A. Lane: 2 Mutagenic Cry1Ia with a triple mutation of R121A+K123A+K129A, Lane 3: Cry1Ia wild-type. Lane 4: Purified Cry1Ia wild-type. The arrow indicates the expected molecular size of Cry1Ia protoxin. Panel b shows the trypsin treatment of Cry1Ia wild-type and R155A+K159A mutant and panel c demonstrates the trypsin treatment of Cry1Ia wild-type and R121A+K123A+K129A mutant. Lanes Wt and Mt show the cell lysates of the *E. coli* cultured cells expressing Cry1Ia wild-type and the *E. coli* cells expressing the mutant proteins before trypsin treatment, respectively. The trypsin digestion of Cry1Ia wild-type and mutant proteins was performed at a ratio of 1:10 [wt/wt] trypsin/protoxin. Samples were analyzed after 1 and 2 hours of treatment, as indicated in the corresponding lanes. Lanes M; molecular mass marker.
